# Hepatic disease and the risk of mortality of *Vibrio vulnificus* necrotizing skin and soft tissue infections: A systematic review and meta-analysis

**DOI:** 10.1371/journal.pone.0223513

**Published:** 2019-10-25

**Authors:** Po-Yao Chuang, Tien-Yu Yang, Tsan-Wen Huang, Yao-Hung Tsai, Kuo-Chin Huang, Hsu-Huei Weng

**Affiliations:** 1 Department of Orthopaedic Surgery, Chiayi Chang Gung Memorial Hospital, Chiayi, Taiwan; 2 Chang Gung University College of Medicine, Taoyuan, Taiwan; 3 Department of Diagnostic Radiology, Chiayi Chang Gung Memorial Hospital, Chiayi, Taiwan; Chulalongkorn University, THAILAND

## Abstract

**Background:**

*Vibrio vulnificus* necrotizing skin and soft tissue infections (VNSSTIs) are associated with a high mortality rate that varies remarkably with host susceptibility. Hepatic disease (HD) is considered the key risk factor for high VNSSTIs incidence and mortality; however, there is limited evidence in the literature to support this observation.

**Methodology:**

We examined all reported cases of VNSSTIs and associated mortality rates between 1966 and mid-2018. The PubMed, Medline and Cochrane Library databases were systematically searched for observational studies on patients with VNSSTIs. Twelve studies with 1157 total patients with VNSSTIs were included in the analysis. From the pooled dataset, nearly half (46.8%) of the patients with VNSSTIs had HD. The mortality rate in HD patients with VNSSTIs was 53.9% (n = 292/542), which was considerably higher than the mortality rate of 16.1% (n = 99/615) in non-HD patients. Patients with HD contracted VNSSTIs were found to be two or more times (RR = 2.61, 95% CI = 2.14–3.19) as likely to die compared with those without HD. Besides, liver cirrhosis (LC), the end-stage HD, was confirmed to be a significant risk factor, with risk ratios of 1.84 (95% CI 1.21–2.79) and 2.00 (95% CI 1.41–2.85) when compared to non-LC and non-HD, respectively.

**Conclusions:**

HD with or without LC can be associated with infections and complications from *V*. *vulnificus*. Clinicians should aggressively approach care and management of acutely and/or critically ill patients with VNSSTIs.

## Introduction

*Vibrio vulnificus* (*V*. *vulnificus*), a naturally occurring Gram-negative bacterium found in estuarine and marine environments throughout the world, is extremely virulent and can cause three types of infections: (1) acute gastroenteritis, (2) primary septicemia, and (3) necrotizing wound infections [1–3]. *V*. *vulnificus* gastroenteritis may be mostly unreported, since it is generally not life-threatening and symptoms are rarely severe to warrant medical intervention [1]. In contrast, patients with primary septicemia or necrotizing wound infections usually develop blistering skin lesions, and are frequently lethal [4]. Often these skin lesions spread rapidly and might involve any layer of the soft tissue compartment associated with widespread necrosis and systemic toxicity [4–6]. Overall, the mortality rate of *V*. *vulnificus* necrotizing skin and soft tissue infections (VNSSTIs) ranges from 30% to 48%; however, the mortality rate varies remarkably according to host susceptibility [1].

Among the risk factors and predisposing conditions, hepatic disease (HD) is considered the key risk factor that increases VNSSTIs incidence and mortality [7–9]; however, there is limited evidence in the literature to support this observation. In the largest series of 310 cases reported by Dechet et al. [10], HD was present in 20% of the patients with VNSSTIs. In contrast, Shapiro et al. [11] performed an analysis on another series of 269 cases and found that more than 50% of these patients had HD. There have been variations in the reported mortality rates in HD patients after contracting VNSSTIs: 23.3% to 66.7% with an average of 53.9% [10–21]. Because of the uncertainty and wide variation in the reported incidence and mortality of VNSSTIs in HD patients, we conducted a systematic review and meta-analysis to determine the risk of mortality of VNSSTIs in patients with HD compared to those without it. Furthermore, we identified studies with additional content mentioning the HD types/satges to explore the effect of HD types/stages on the risk of mortality of VNSSTIs. The information from this study may be valuable for clarifying the relationship between HD and the risk of mortality of VNSSTIs, thereby facilitating future research and then reducing the mortality rate.

## Materials and methods

### Search strategy and selection criteria

This study was approved by the ethics committee (Institutional Review Board) of the Chang Gung Memorial Hospital in Taiwan (reference number: 103-7038C). A protocol was developed in advance of conducting this systematic review and meta-analysis according to the Meta-Analysis of Observational Studies in Epidemiology (MOOSE) criteria [22] and the Preferred Reporting Items for Systematic Review and Meta-Analysis (PRISMA) checklist (S1 Checklist). To identify studies assessing the mortality of VNSSTIs, we searched for publications in PubMed, Medline and Cochrane Library database between January 1966 and July 2018 without restrictions on year of publication. The combination of key words (free text and controlled vocabulary terms) in the search strategy included “Vibrio vulnificus*”, “infect*”, “death”, “mortality”, and “fatality”. We also reviewed manually the references cited in articles that were retrieved. No language restrictions were placed on the searches or search results.

Our first question focused on the risk of mortality of VNSSTIs in patients with HD compared to those without it. Eligible studies were observational studies that had one group of patients with HD and another group of patients without it. The second question was on the effect of HD types/stages on the risk of mortality of VNSSTIs. The eligible studies were those articles with additional content mentioning the HD types/stages. Identified studies were reviewed for eligibility by two authors (PYC and TYY) based first on the title, then the abstract and then finally on the full study.

### Quality assessment and data extraction

Included studies were assessed for quality using the Epidemiological Appraisal Instrument (EAI) [23]. Title, author and journal details were removed to de-identify articles prior to rating. Two authors (TWH and YHT) completed the assessment of quality, and disputes were resolved by discussion with a third author (KCH or HHW). For each study, data extraction was completed using a pre-designed data extraction form. We abstracted data on average age, sex, presence and types/stages of HD, and the number of patients who survived or perished from VNSSTIs. Disagreements on quality assessment and data extraction were resolved by consensus and if none was arrived at, by discussion with others in regular meetings.

### Data analysis and statistical methods

RevMan 5.3 software (Cochrane Collaboration, Oxford, UK) was used in this meta-analysis. The risk ratios estimating the risk of mortality of VNSSTIs in patients with HD compared to those without it of the individual studies were combined using the Mantel-Haenszel method. Heterogeneity across studies was assessed by the I^2^ and χ^2^ tests. We planned to use the Random Effects Model (REM) instead of Fixed Effects Model (FEM) if the I^2^ ≥ 40%. In the pre-specified subgroup analyses we estimated the risk ratios of mortality of VNSSTIs in the following populations: (1) studies conducted in the subtropical western Atlantic or Pacific coastal areas, and (2) studies showing the HD types/stages like hepatitis and liver cirrhosis (LC), among others. Publication bias was assessed using methods based on the funnel plot, such as Begg’s test and Egger’s test.

### Ethic statement

The data were analyzed after approval by the ethics committee (Institutional Review Board) of the Chang Gung Memorial Hospital in Taiwan (reference number: 103-7038C). All data analyzed were anonymized.

## Results

**Fig 1:**
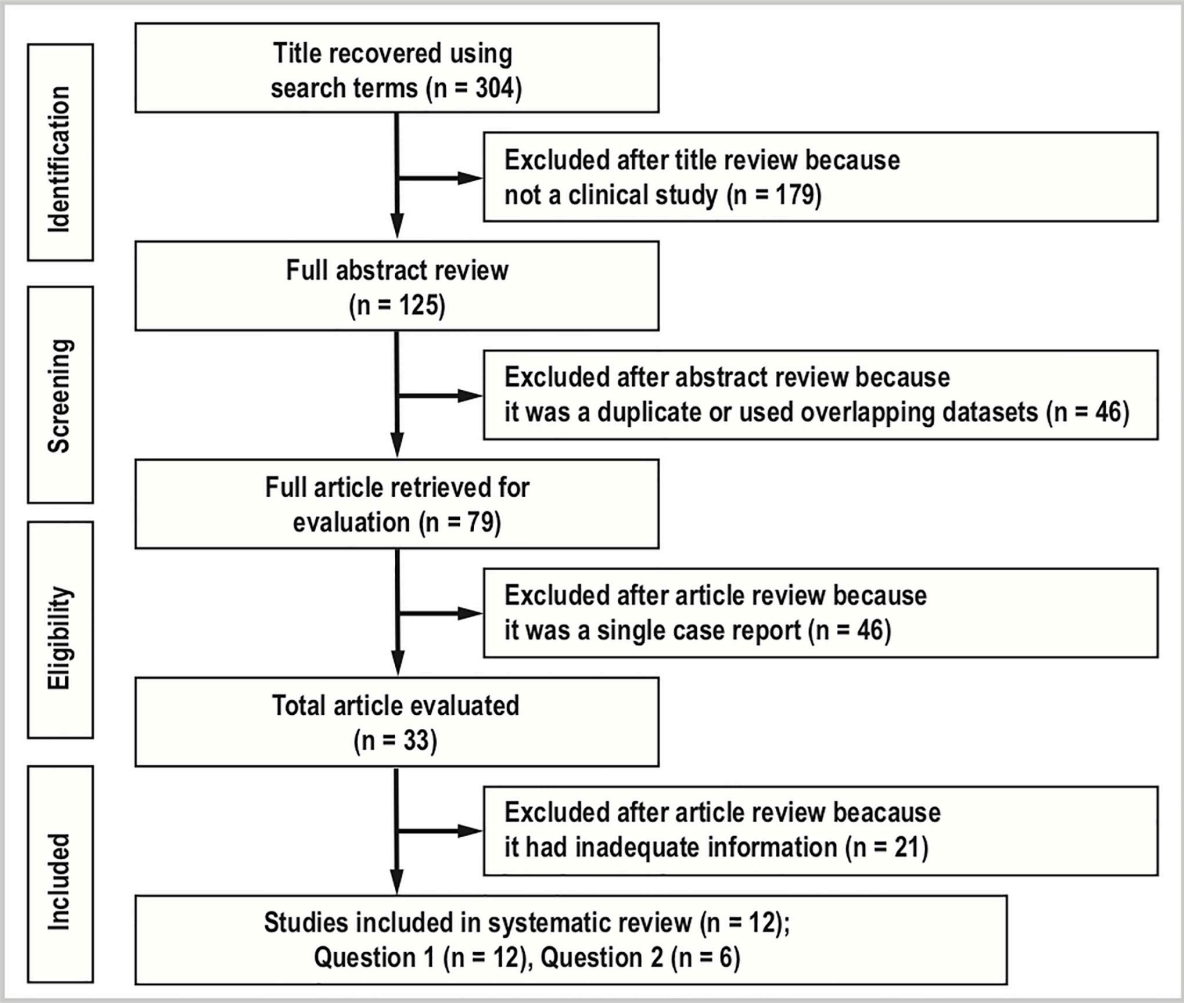
PRISMA flow diagram of selection process of eligible studies. The figures indicate the number of articles reviewed at each stage.

Fig 1 summarizes the selection process of studies and shows the number of articles included in the review stages. As a result of electronic and manual searches, we examined a total of 304 article titles and their abstracts. Of these, we excluded 179 articles that were not clinical studies and 46 that were duplicates or used overlapping datasets. An additional 67 articles were excluded after full text review, with the main reasons for rejection being lack of detail regarding the comorbid HD to answer the questions of this study. Ultimately, we identified 12 and 6 studies that fulfilled the eligibility criteria for the systematic review for questions 1 and 2, respectively. The risk of publication bias is shown in the funnel plot (S1 Fig), which does not suggest significant publication bias.

**Fig 2:**
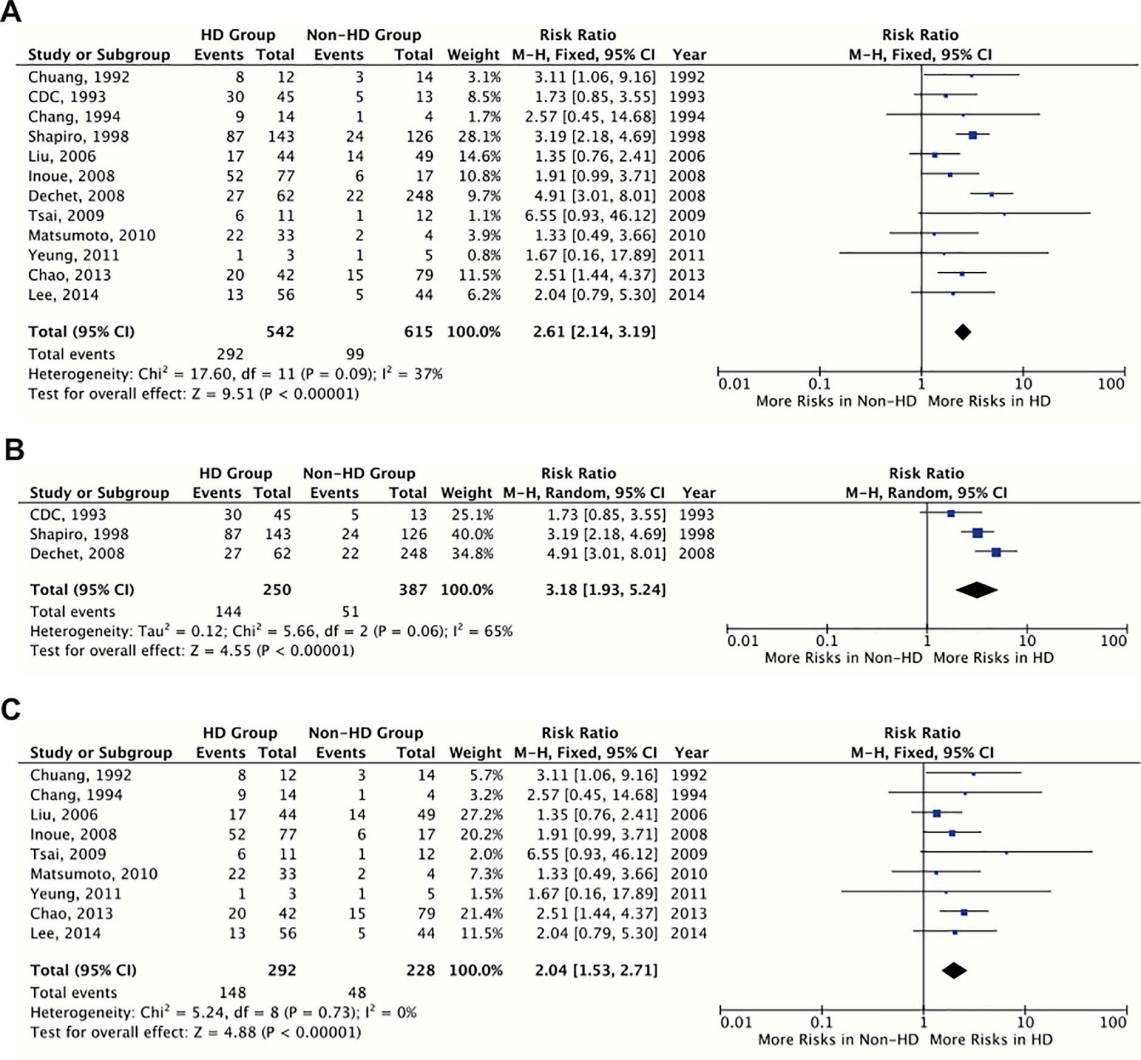
**Risk of mortality of *Vibrio vulnificus* necrotizing skin and soft tissue infections in patients with hepatic disease compared to those without it: (A) the world’s coastal areas; (B) the western Atlantic coastal areas; and (C) the western Pacific coastal areas.** “Study or subgroup” on the Y-axis refers to first author and publication year. “Events” refers to the number of patients who died. “Total” refers to the number of patients in that group. “Weight” refers to the influence of each study on overall estimate (weights are from fixed effect analyses for I^2^ < 40% and random effect analyses for I^2^ ≥ 40%). For each study the central square indicates risk ratio, the line represents the 95% confidence interval (CI), and the size of the square reflects the study’s weight in the pooling. “Overall estimate” refers to pooled estimate of risk ratio after mathematical combination of all studies. The X-axis indicates the scale and the direction of the effect of hepatic disease on the risk of mortality. I-squared denotes the extent of heterogeneity in study outcomes, with a hypothetical value of 100% meaning considerable heterogeneity and 0% meaning no heterogeneity between studies.

The characteristics of the studies included in the review are detailed in S1 Table. The total number of patients with HD was 542 (46.8%) and there were 615 (53.2%) non-HD patients. The overall pooled proportion of individuals who died in the HD group was 53.9% (n = 292/542) while in the non-HD group 16.1% (n = 99/615) of the individuals died after contracting VNSSTIs. Patients with HD had more risk of mortality compared to those without it with a risk ratio (RR) of 2.61 (95% CI 2.14–3.19) (Fig 2A). In a subgroup analysis, we reviewed studies done in the subtropical western Atlantic (n = 3) and Pacific (n = 9) coastal areas. The overall RRs of mortality after contracting VNSSTIs in patients with HD compared to those without it were 3.18 (95% CI 1.93–5.24, n = 637) for those living in the subtropical western Atlantic coastal areas and 2.04 (95% CI 1.53–2.71, n = 520) for those living in the subtropical western Pacific coastal areas (Fig 2B and 2C).

**Fig 3:**
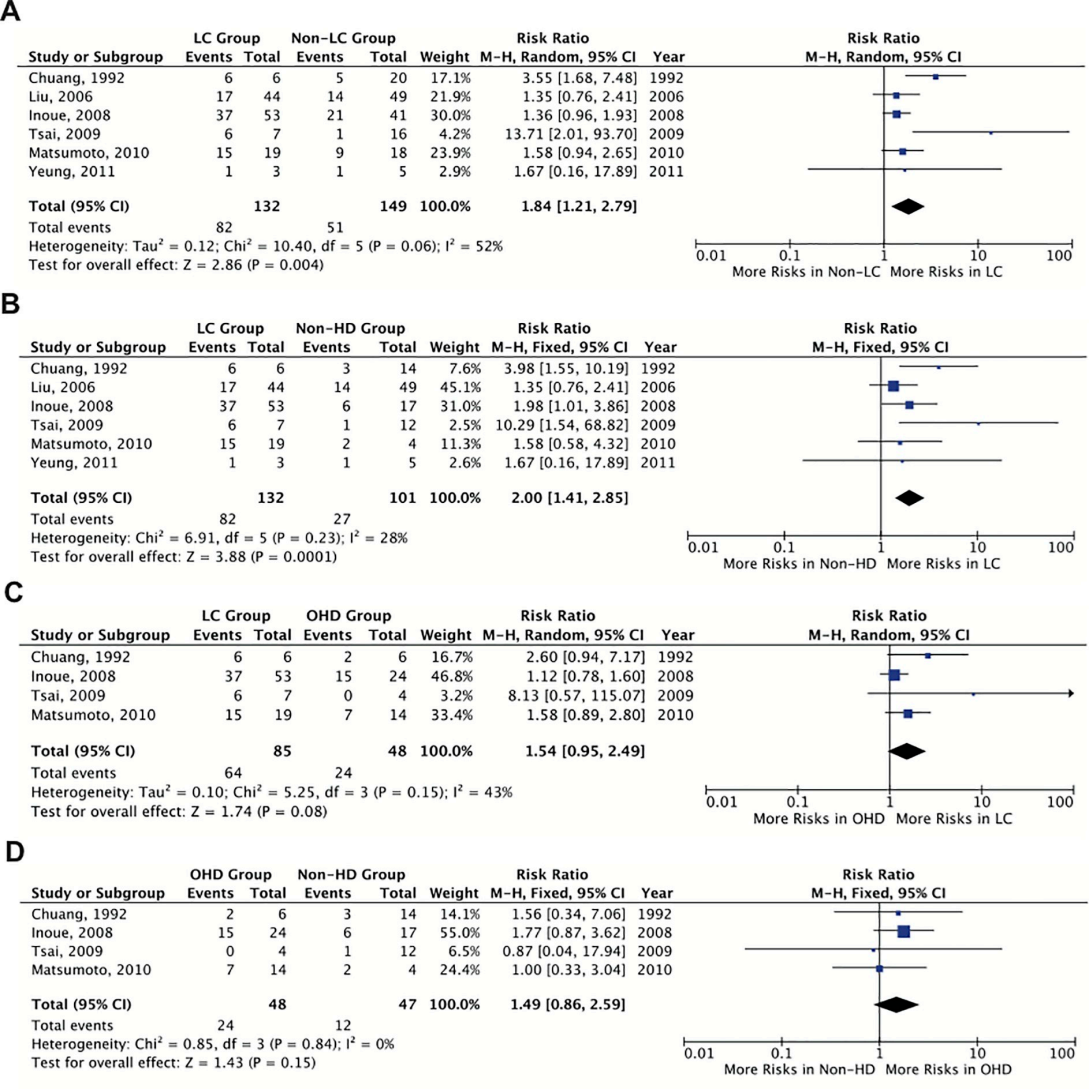
**Risk of mortality of *Vibrio vulnificus* necrotizing skin and soft tissue infections in patients with liver cirrhosis (LC) compared to the controls: (A) the LC group vs. the non-LC group; (B) the LC group vs. the non-hepatic disease (HD) group; (C) the LC group vs. the other hepatic disease (OHD) group; and (D) the OHD group vs. the non-HD group.** “Study or subgroup” on the Y-axis refers to first author and publication year. “Events” refers to the number of patients who died. “Total” refers to the number of patients in that group. “Weight” refers to the influence of each study on overall estimate (weights are from fixed effect analyses for I^2^ < 40% and random effect analyses for I^2^ ≥ 40%). For each study the central square indicates risk ratio, the line represents the 95% confidence interval (CI), and the size of the square reflects the study’s weight in the pooling. “Overall estimate” refers to pooled estimate of risk ratio after mathematical combination of all studies. The X-axis indicates the scale and the direction of the effect of LC on the risk of mortality. I-squared denotes the extent of heterogeneity in study outcomes, with a hypothetical value of 100% meaning considerable heterogeneity and 0% meaning no heterogeneity between studies.

Data from 6 studies containing additional information on the HD types/stages were extracted for exploring their effects on the risk of mortality of VNSSTIs. The total number of patients with LC was 132 (47.0%) and there were 149 (53.0%) non-LC and 101 (35.9%) non-HD patients. The overall pooled proportion of individuals who died in the non-LC and non-HD groups were 34.2% and 26.7% while in the LC group 62.1% of the individuals died after contracting VNSSTIs. Patients with LC had an almost two fold higher risk (n = 82/132) of mortality compared to those without LC (n = 51/149) and HD (n = 27/101) with RRs of 1.84 (95% CI 1.21–2.79) and 2.00 (95% CI 1.41–2.85), respectively (Fig 3A and 3B). Increased risks of mortality in the LC group *vs*. the other HD (OHD) group and the OHD group *vs*. the non-HD group was also observed; however, they were not statistically significant (RR: 1.54, 95% CI 0.95–2.49, *p* = 0.08; RR: 1.49, 95% CI 0.86–2.59, *p* = 0.15) (Fig 3C and 3D).

## Discussion

Through this systematic review and meta-analysis, we confirmed that HD is a key host susceptibility risk factor that increases VNSSTIs incidence and mortality. From the pooled dataset, nearly half (46.8%) of the patients with VNSSTIs had HD. In addition, the mortality rate in HD patients with VNSSTIs was 53.9%, which was considerably higher than 16.1% of non-HD patients. When patients with HD contracted VNSSTIs, they were twice as likely (RR = 2.61, 95% CI = 2.14–3.19) to die compared with those without HD. Among the types/stages of HD, LC is confirmed to be a significant risk factor, with RRs of 1.84 (95% CI 1.21–2.79) and 2.00 (95% CI 1.41–2.85) comparing non-LC and non-HD, respectively. These findings highlight that HD with or without LC can be associated with infections and complications from *V*. *vulnificus* and clinicians should pay attention to them and be aggressive when approaching and caring for these acutely and/or critically ill patients.

There are more than 100 known types of HD caused by a variety of factors and affecting everyone from infants to older adults. Among them, viral and alcoholic hepatitis are the most common forms of HD [24]. Although there is a wide range of HD types, the stages and damage to the liver are usually consistent. They progress through a series of four main stages of hepatic damage: inflammation, fibrosis, LC and liver failure [25]. The onset of hepatic fibrosis is usually insidious, and advanced hepatic fibrosis will result in LC [26]. The concept of LC has changed from being a static and irreversible entity to a dynamic disease with reversible stages [27,28]. Nonetheless, LC with or without decompensation is still a critical stage associated with a variety of major complications [29]. Acute-on-chronic liver failure (AoCLF) is an increasingly recognized distinct disease which encompasses an acute deterioration in liver function, hepatic and extra-hepatic organ failures, and an association with substantial short-term mortality [30]. Its frequency and severity increase as hepatic functional reserve (HFR) worsens, and common precipitants include bacterial and viral infections, alcoholism, and surgery [30,31]. Continued efforts have been made to develop a predictive scoring system for assessment of HFR and as a predictor and monitor of short-term prognosis of cirrhotic patients undergoing surgery or contracting severe infections, such as VNSSTIs [32,33].

As shown in this study, HD with or without LC can be associated with infections and complications from *V*. *vulnificus*. Liver dysfunction may play a key role in the pathogenesis of these infections. The liver has a role in bacteria and endotoxin scavenging, detoxication, and synthesizing proteins for metabolic, immune, and coagulation functions. Liver dysfunction seems to affect the susceptibility and prognosis of VNSSTIs in multiple ways. Bacterial and endotoxin clearance was impaired in those with underlying HD, which could explain a higher susceptibility of the host to infection [34,35]. Increased cellular oxidative stress is common in HD and related to cytokine dysfunction, which contributes to the increased risk of *V*. *vulnificus* septicemia and may result in more complications [36]. The bioactivity of tumor necrosis factor-α (TNF-α) was found to be significantly lower in cirrhotic mice compared with non-cirrhotic mice, and the mortality rate in cirrhotic mice was significantly higher but could be reversed by pretreatment with TNF-α [37]. *V*. *vulnificus* possesses multiple iron-regulated genes and appears to have more virulence in environments with high levels of available iron. Therefore, high levels of serum iron in HD, due to disrupted iron physiology, may also affect neutrophil activity and enhance the survival of *V*. *vulnificus* in the blood [38,39]. In addition, C-reactive protein (CRP), a typical hepatogenic acute phase protein, was noted to play an important role in the protection of animals from lethality induced by *V*. *vulnificus* infection, however their production was impaired due to preexisting hepatic dysfunction [40].

Interestingly, HD patients living in the subtropical western Atlantic coastal areas had a higher RR of mortality compared to those in the subtropical western Pacific coastal areas (3.18 *vs*. 2.04). This mortality discrepancy may be due to racial differences; however, we need more direct evidence to support this point. Moreover, we observed that the distribution of the causes of LC were different in these places due to variation in the endemic prevalence of viral hepatitis and ethnic differences in alcohol consumption [41]. For example, the estimated fractions of LC s to HBV infection ranged from 5% in USA, 14% in Japan to 57% in China, south Korea, and Taiwan. In contrast, the fractions of LC attributable to HCV infection ranged from 62% in Japan, 42% in USA to 21% in China, south Korea and Taiwan [41]. The differences in the distribution of LC causes may contribute to the discrepancy in geographical mortality due to VNSSTIs. Alternatively, there are marked differences in the antibiotic resistance profile of *V*. *vulnificus* worldwide [42]. Although Infectious Diseases Society of America (ISDA) and CDC suggest doxycycline with ceftazidime, ceftriaxone or cefotaxime as the first-line regimen in adults with VNSSTIs, antimicrobial agents should be tailored in different countries [42]. For example, doxycycline has shown intermediate resistant profile in Italy [43] while ceftazidime in the U.S. [44] and ceftriaxone in India [45]. There is inconsistency in surgical approaches for this kind of severe soft tissue infection, particularly amongst different regions due to the low prevalence of this disease and lack of literature. Further detailed clinical studies and focused education is needed to improve the outcome of treatment and to decrease the associated high mortality rate.

In summary, VNSSTIs is an important public health problem and is becoming more critical because of global warming [46]. The stages of HD strongly correlated with the mortality rates after VNSSTIs. A discrepancy exists between the mortality rate in subtropical western Atlantic coastal areas and western Pacific coastal areas. Further studies to understand and clarify the risk factors or mechanisms of disease, controlling and/or reversing them, and finding a clinical pathway that can lower the mortality are crucial.

## Supporting information

S1 Checklist PRISMA checklist. (PDF)

S1 Table Included studies of *Vibrio vulnificus* necrotizing skin and soft tissue infections (VNSSTIs). (DOCX)

S1 Fig Funnel plot of studies estimating the risk ratio of mortality of *Vibrio vulnificus* necrotizing skin and soft tissue infections in patients with hepatic disease compared to those without it. Points indicate the risk ratios (X-axis) from 12 studies assessing the risk of mortality of *Vibrio vulnificus* necrotizing skin and soft tissue infections in patients with hepatic disease when compared to those without it.
(TIFF)

S2 Fig Bubble plot with fitted meta-regression line of the log mortality of *Vibrio vulnificus* necrotizing skin and soft tissue infections, 12 studies published between 1990 and 2015. Each circle represents a study in the meta-analysis, and the size of the circle is proportional to study weighting.
(TIFF)
