# Supplementary material for: Hepatic disease and the risk of mortality of Vibrio vulnificus necrotizing skin and soft tissue infections: A systematic review and meta-analysis
Source: PLoS One. 2019 Oct 25;14(10):e0223513. doi: 10.1371/journal.pone.0223513 (PMC6814278; doi:10.1371/journal.pone.0223513)
Supplement: S1 Table — (DOCX) [file pone.0223513.s002.docx]

**S1 Table.** **Included studies of *Vibrio vulnificus* necrotizing skin and soft tissue infections (VNSSTIs)**

| **First Author,** | **Country** | **Age** | | **Male Sex** | **Case** | **Death** | **Mortality (%)** | | | | |
| --- | --- | --- | --- | --- | --- | --- | --- | --- | --- | --- | --- |
| **Publication year** | **Conducted** | **Mean** | **Spread** | **(%)** | **No.** | **No.** | **Total** | **HD^§^** | **Non-HD** | **LC^¶^** | **Non-LC** |
| Chuang et al. 1992 | Taiwan | 58.7 | 22-76 | 73.1 | 26 | 11 | 42.3 | 66.7 | 21.4 | 100.0 | 25.0 |
| CDC. 1993 | USA | 60.0 | 33-90 | 87.9 | 107 | 44 | 41.1 | 66.7 | 38.5 | NA | NA |
| Chang et al. 1994 | Taiwan | NA | NA | NA | 18 | 10 | 55.6 | 64.3 | 25.0 | NA | NA |
| Shapiro et al. 1998 | USA | 56.6 | 4-92 | 88.4 | 370 | 142 | 38.4 | 60.8 | 19.0 | NA | NA |
| Liu et al. 2006 | Taiwan | 62.2 | 9-87 | 72.0 | 93 | 31 | 33.3 | 38.6 | 28.6 | 38.6 | 28.6 |
| Dechet et al. 2008^26^ | USA | 63.0 | 1-94 | 88.7 | 375 | 62 | 16.5 | 44 | 9 | NA | NA |
| Inoue et al. 2008 | Japan | 60.6 | 1-81 | 83.0 | 94 | 58 | 61.7 | 67.5 | 35.3 | 69.8 | 51.2 |
| Tsai et al. 2009 | Taiwan | 60.6 | 36-78 | 73.9 | 23 | 7 | 30.4 | 54.5 | 8.3 | 85.7 | 6.3 |
| Matsumoto et al. 2010 | Japan | 59.9 | 30-94 | 94.6 | 37 | 24 | 64.9 | 66.7 | 50.0 | 78.9 | 50 |
| Yeung et al. 2011 | HK | NA | NA | NA | 8 | 2 | 25.0 | 33.3 | 20.0 | 33.3 | 20 |
| Chao et al. 2013 | Taiwan | 65.2 | 29-89 | 62.0 | 121 | 35 | 28.9 | 47.6 | 19.0 | NA | NA |
| Lee et al. 2014 | Taiwan | 63.6 | 10-90 | 79.0 | 100 | 18 | 18.0 | 23.2 | 11.4 | NA | NA |

§ HD, hepatic disease. (Note: detailed types not available in the original articles)

¶ LC, liver cirrhosis. (Note: definition criteria and etiology not available in the original articles)
