# Supplementary figures and images for: Hepatic disease and the risk of mortality of Vibrio vulnificus necrotizing skin and soft tissue infections: A systematic review and meta-analysis
Source: PLoS One. 2019 Oct 25;14(10):e0223513. doi: 10.1371/journal.pone.0223513 (PMC6814278; doi:10.1371/journal.pone.0223513)

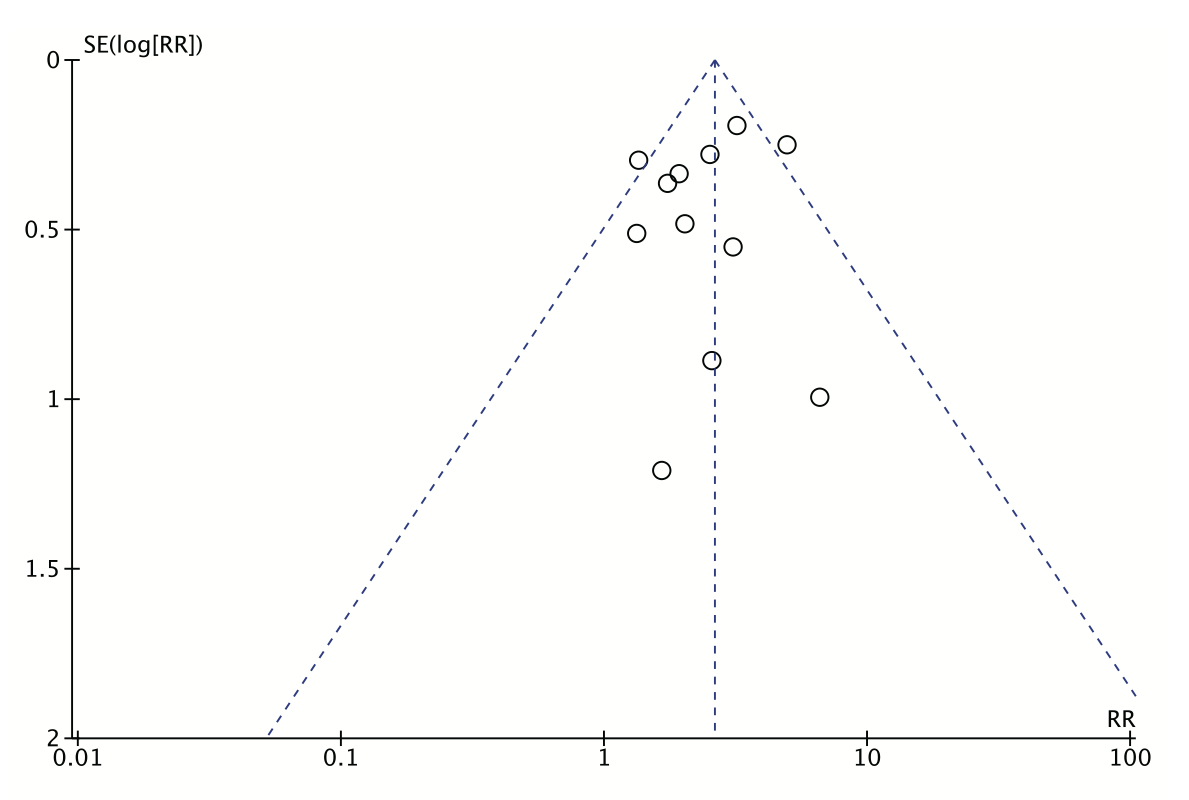

Supplement: S1 Fig — Points indicate the risk ratios (X-axis) from 12 studies assessing the risk of mortality of Vibrio vulnificus necrotizing skin and soft tissue infections in patients with hepatic disease when compared to those without it. (TIFF) [file pone.0223513.s003.tiff]
